# Identification of deposits from modern and ancient large tsunamis by means of environmental DNA

**DOI:** 10.1038/s41598-024-84245-y

**Published:** 2025-01-02

**Authors:** Tetsuya Shinozaki, Akira Iguchi, Miyuki Nishijima, Kazuhisa Goto, Shigehiro Fujino

**Affiliations:** 1 Department of Earth and Planetary Science, The University of Tokyo, 7-3-1 Hongo, Bunkyo-ku, Tokyo, 113-0033 Japan; 2 Geological Survey of Japan, National Institute of Advanced Industrial Science and Technology (AIST), 1-1-1 Higashi, Tsukuba, Ibaraki 305-8567 Japan; 3 Research Laboratory On Environmentally-Conscious Developments and Technologies [E-Code], National Institute of Advanced Industrial Science and Technology (AIST), 1-1-1 Higashi, Tsukuba, Ibaraki 305-8567 Japan; 4 Faculty of Life and Environmental Sciences, University of Tsukuba, 1-1-1 Tennodai, Tsukuba, Ibaraki 305-8572 Japan

**Keywords:** Tsunami deposit, Environmental DNA, 2011 Tohoku-oki tsunami, Historical and prehistoric event, Natural hazards, Ocean sciences

## Abstract

We examined the potential of environmental DNA (eDNA) for identifying tsunami deposits in the geological record using lake-bottom sediments in the Tohoku region, Japan. The presence of eDNA from marine organisms in a lacustrine event deposit provides very strong evidence that the deposit was formed by an influx of water from the ocean. The diverse DNA assemblage in the deposit formed by the 2011 Tohoku-oki tsunami included DNA of marine origin indicating that eDNA has potential as an identifying proxy for tsunami deposits. Subsequently, we examined the applicability of eDNA for recognizing paleo-tsunami events using the deposits formed by the 869 CE Jogan tsunami and a prehistoric event (2400–2900 cal year BP). The taxa detected in the tsunami deposits were markedly different from those of the background sediments. Many taxa that were represented in the Jogan tsunami deposit were also detected in the layer immediately above the tsunami deposit. This layer was indistinguishable from the overlying peat by visual observation, but the eDNA results suggest that it is likely to be a muddy tsunami deposit. The results of this study indicate that eDNA has the potential to elucidate the origin of event deposits that have been difficult to identify.

## Introduction

A tsunami, which is an abrupt and high-energy event, brings allochthonous sediments such as beach and seafloor sand to terrestrial areas; these sediments, which are called tsunami deposits, are easily recognized as event layers in geological strata. The occurrence age of a past tsunami can be estimated by examining the depositional age of the associated tsunami deposits. The distribution area of tsunami deposits indicates at least the minimum inundation area of a tsunami, and the tsunami magnitude can be estimated by numerical simulation. Thus, the recurrence interval and magnitude of paleo-tsunamis can be estimated by examining the depositional age and distribution of tsunami deposits^1–7^, and tsunami disaster risk assessment has been conducted based on reconstructed paleo-tsunami information^8^.

The identification of tsunami deposits in the geological record is based on the occurrence of sedimentological features such as structures formed by rapid flow, micropaleontological features such as the presence of marine micro-organisms, and geochemical features such as changes in the concentration of seawater components^9–12^. However, these features can be lost over time and are not necessarily retained in the geological record. In addition, storm surges and floods also transport allochthonous sediments, potentially giving rise to confusion between deposits left by these events and by tsunamis^13^. Evidence used in the past to identify tsunami deposits was often based on indirect indicators, so there is an urgent need to find proxies that provide direct evidence of tsunami inflow.

In this study, we focused on environmental DNA (eDNA) as a proxy for identifying tsunami deposits. The presence of genetic information of marine organisms in an event deposited on land provides strong evidence that it was transported by flow from the ocean. Several studies applying genetic analysis to tsunami deposits have been conducted in the last decade^14–18^. In particular, Yap et al.^17^ conducted eDNA analyses of sediments from a modern tsunami, the 2004 Indian Ocean tsunami, and subsequent storm flooding events, the 2011 Cyclone Thane at Cuddalore, India, and the 2007 storm at Phra Thong Island, Thailand. They noted that the microbial communities contained in the sediments from each event were different, which allowed discrimination of sediments deposited during the tsunami and flood events. Yap et al.^18^ collected samples from the 2004 Indian Ocean tsunami and paleo-tsunamis that occurred approximately 550–700 years ago and around 2800 years ago from a trench excavated in a coastal wetland in western Thailand. They reported that the microbial communities contained in the deposits of the 2004 Indian Ocean tsunami and those of the paleo-tsunami 550–700 years ago were different from those of the background sediment. However, the paleo-tsunami deposit from about 2800 years ago was not distinguishable from the overlying and underlying background sediments based on the microbial communities: this lack of difference was attributed to the fact that the event layer is constantly saturated by groundwater. Thus, the preservation of eDNA and its behavior in different depositional environments are still under investigation. In the present study, we performed eDNA analysis on lake-bottom sediments continuously at a thickness of 1 cm. We assessed the validity of our research approach by using the 2011 Tohoku-oki tsunami deposit and then extended the work to consider the applicability of eDNA to paleo-tsunamis for the Jogan tsunami of 869 CE and a prehistoric event that occurred approximately 2400 to 2900 years ago.

**Fig. 1:**
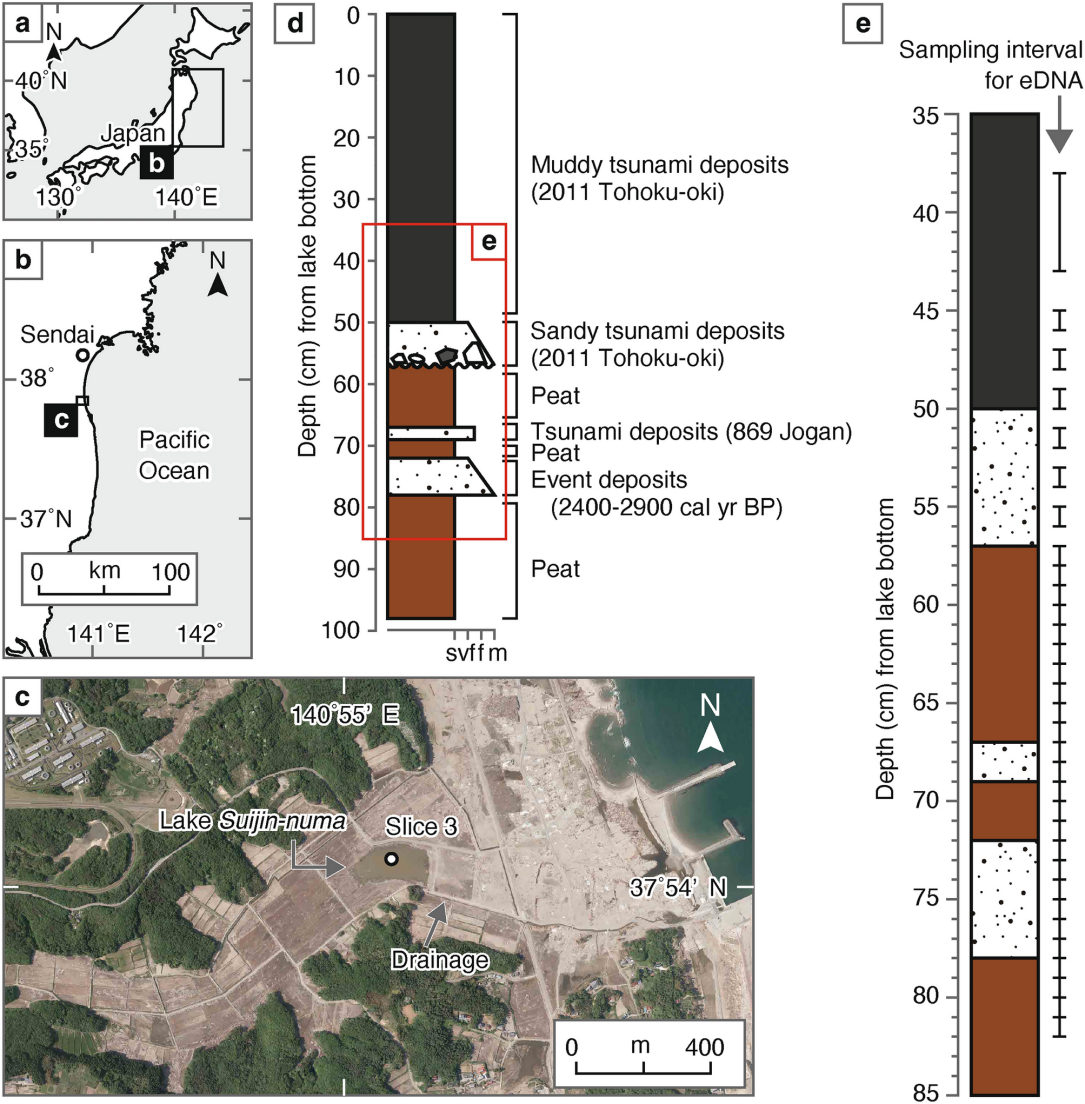
Location maps of the study site and lithologic profiles. (**a**) Map of Japan. (**b**) Map of East Japan. (**c**) Close-up image of the study area. The aerial photograph is from the geospatial information authority of Japan (CTO-2011-10-W-C22_0016 and _0017) and was taken in October 2011. (**d**) Lithologic profile of Slice 3 obtained from the lake center. (**e**) Close-up of the lithologic profile of Slice 3, showing the sampling intervals for eDNA analysis.

The study site is Lake *Suijin-numa*, a small lake (~ 600 m inland from the modern shoreline) located ca. 40 km south of Sendai City, Miyagi Prefecture (Fig. 1a–c). The lake does not receive major river input but it does have narrow drainage (Fig. 1c). Lake *Suijin-numa* is recognized to have been a lake before the 2011 Tohoku-oki tsunami from aerial photographs. Research on tsunami deposits has been conducted in the area before and after the 2011 Tohoku-oki tsunami^19–21^. A 98-cm-long sediment sample obtained in April 2014 from the center of the lake using a 3-m-long handy-geoslicer^22,23^, named Slice 3, was used in our analysis (Fig. 1c). The 2011 Tohoku-oki tsunami deposit was identified by Shinozaki et al.^20^ using sedimentological and diatom analysis. The tsunami deposit consists of a muddy layer from the lake bottom to 50 cm depth underlain by a 7-cm-thick very fine to medium-grained sandy layer (Fig. 1d). The sediment below the 2011 Tohoku-oki tsunami deposit is composed of peat, which is thought to have been formed under background environmental conditions. Two sandy event layers are intercalated within the peat (Fig. 1d). Based on ^14^C dating, the upper and lower sand layers have been inferred to have been deposited by the 869 CE Jogan tsunami and a prehistoric event that occurred at approximately 2400 to 2900 years ago, respectively^20^.

## Results and discussion

### Verification of the research approach using sediments from the 2011 Tohoku-oki tsunami

In total, eDNA measurements were conducted on 32 layers in Slice 3 (Figs. 1e and S1). First, we examined whether there were differences in eDNA between the tsunami deposit and the background sediments by using the results of the eDNA analysis of the muddy (38–50 cm depth) and sandy (50–57 cm depth) parts of the deposit formed by the 2011 Tohoku-oki tsunami and the underlying peat deposited under background conditions (57–67 cm depth).

**Fig. 2:**
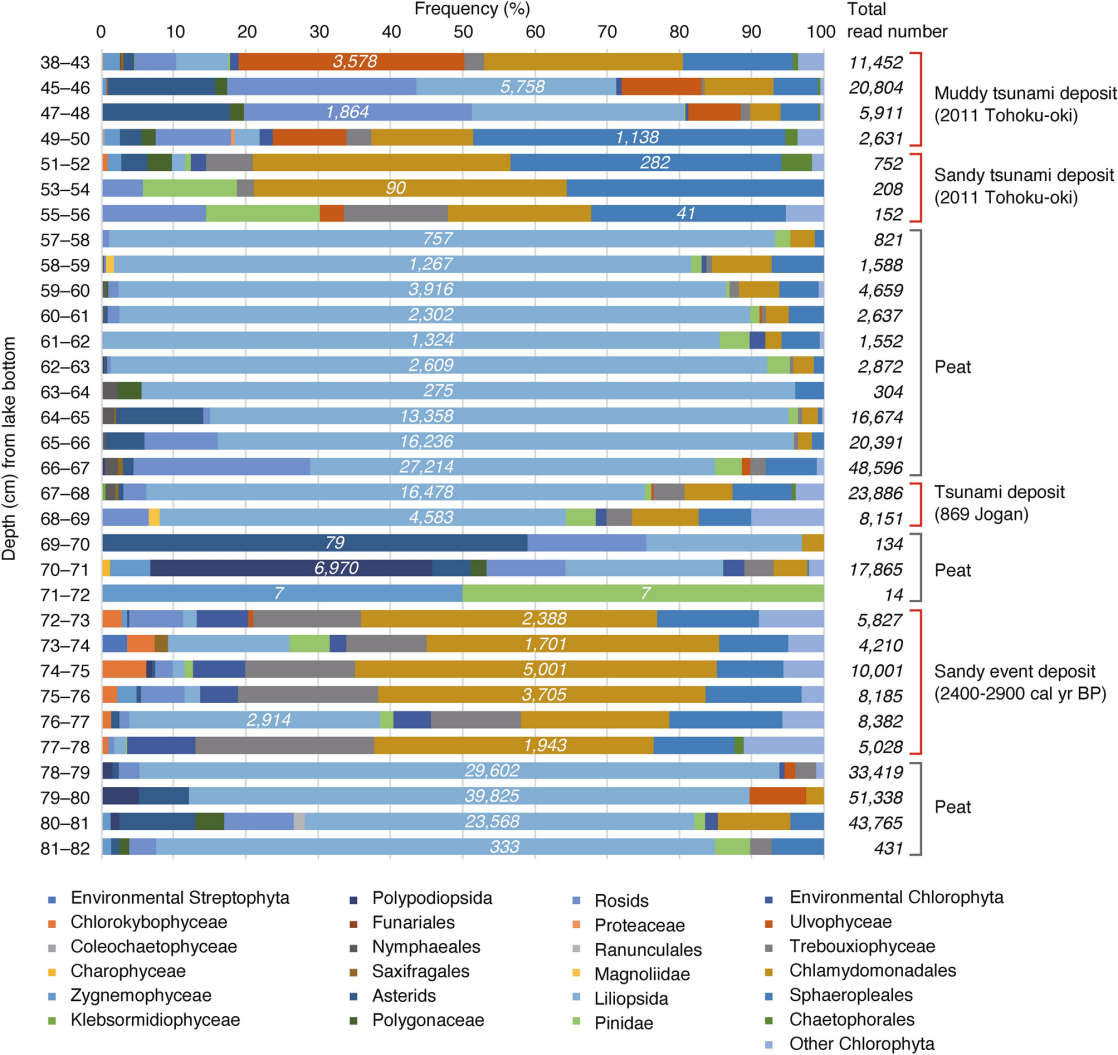
Relative abundances and total read number of *Viridiplantae* DNA sequences in each sampled layer in Slice 3. Numbers in italics are the number of reads of the most abundant DNA in each depth.

The majority of the *Viridiplantae* (subkingdom) sequences represented taxa from terrestrial or freshwater environments (Fig. 2, Table S1). *Liliopsida* (class) was dominant in the peat during background deposition; in contrast, in the muddy tsunami deposit, a wide variety of DNA sequences, including *Ulvophyceae* (class), *Pinidae* (subclass), *Liliopsida* (class), *Asteridaes* (subclass), and *Rosids* (clade), were detected. The sandy tsunami deposit showed the same DNA diversity as the muddy tsunami deposit and included *Chlamydomonadales* (order), *Sphaeropleales* (order), and *Pinidae* (subclass) (Fig. 2, Table S1).

**Fig. 3:**
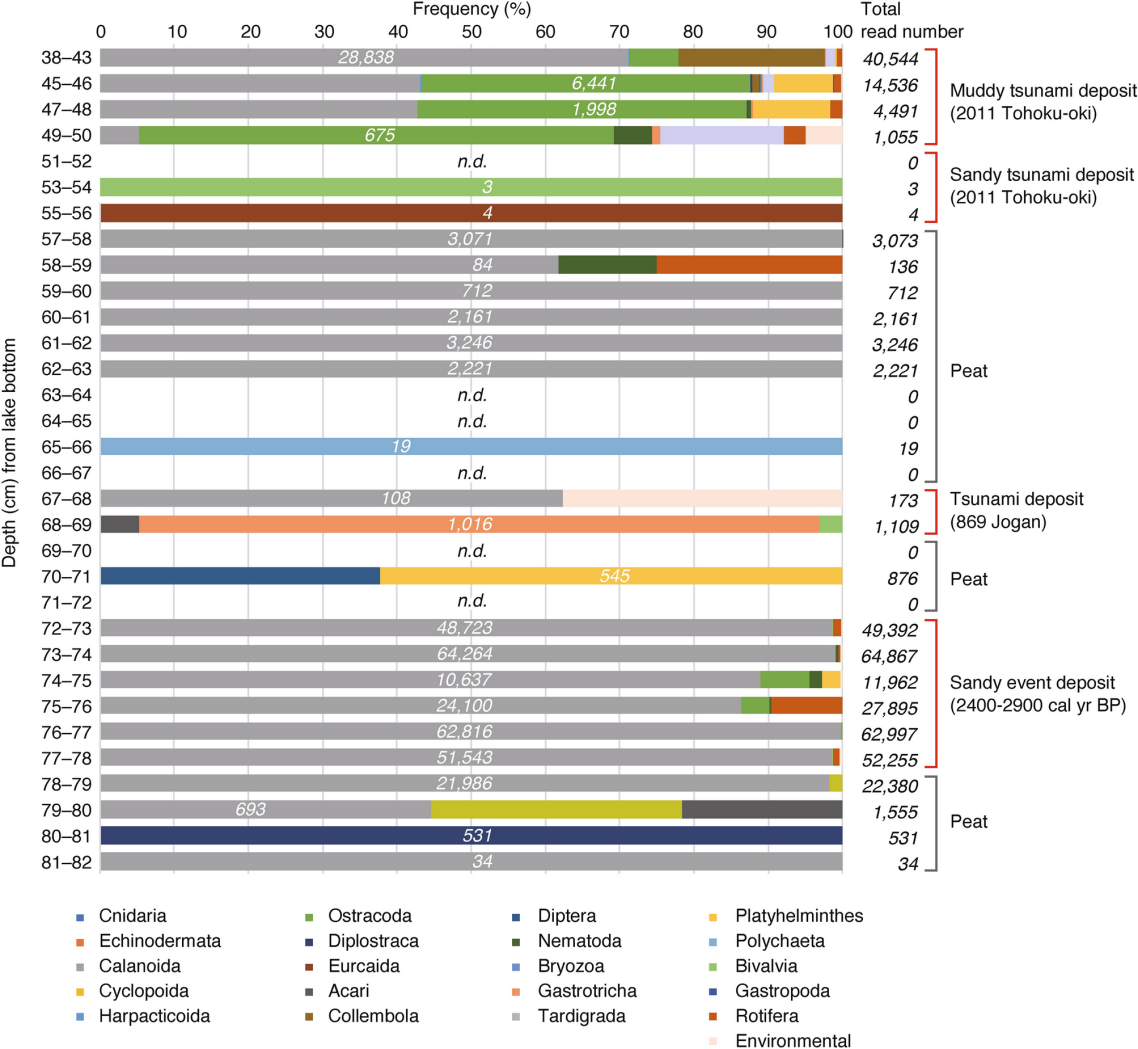
Relative abundances and total read number of *Metazoa* DNA sequences in each sampled layer in Slice 3. Numbers in italics are the number of reads of the most abundant DNA in each depth. *n.d.* not detected.

For *Metazoa*, the number of detected taxa was limited, with only *Calanoida* being detected in many of the peat layers; in contrast, in the muddy part of the deposit, various gene sequences including *Copepoda*, *Ostracoda*, and *Platyhelminthes* were detected (Fig. 3, Table S2). Preservation of DNA in the sandy tsunami sediment was lower than that in the muddy part of the tsunami deposit. The number of DNA reads was on the order of 10^3^ for the muddy part of the tsunami deposit, but in single digits for the sandy tsunami deposit (Fig. 3, Table S2). A small amount of oyster DNA was detected at 53–54 cm depth. The DNA obtained was mostly composed of sequences from unidentified species and golden algae but small amounts of DNA from crustaceans and mollusks were detected.

**Fig. 4:**
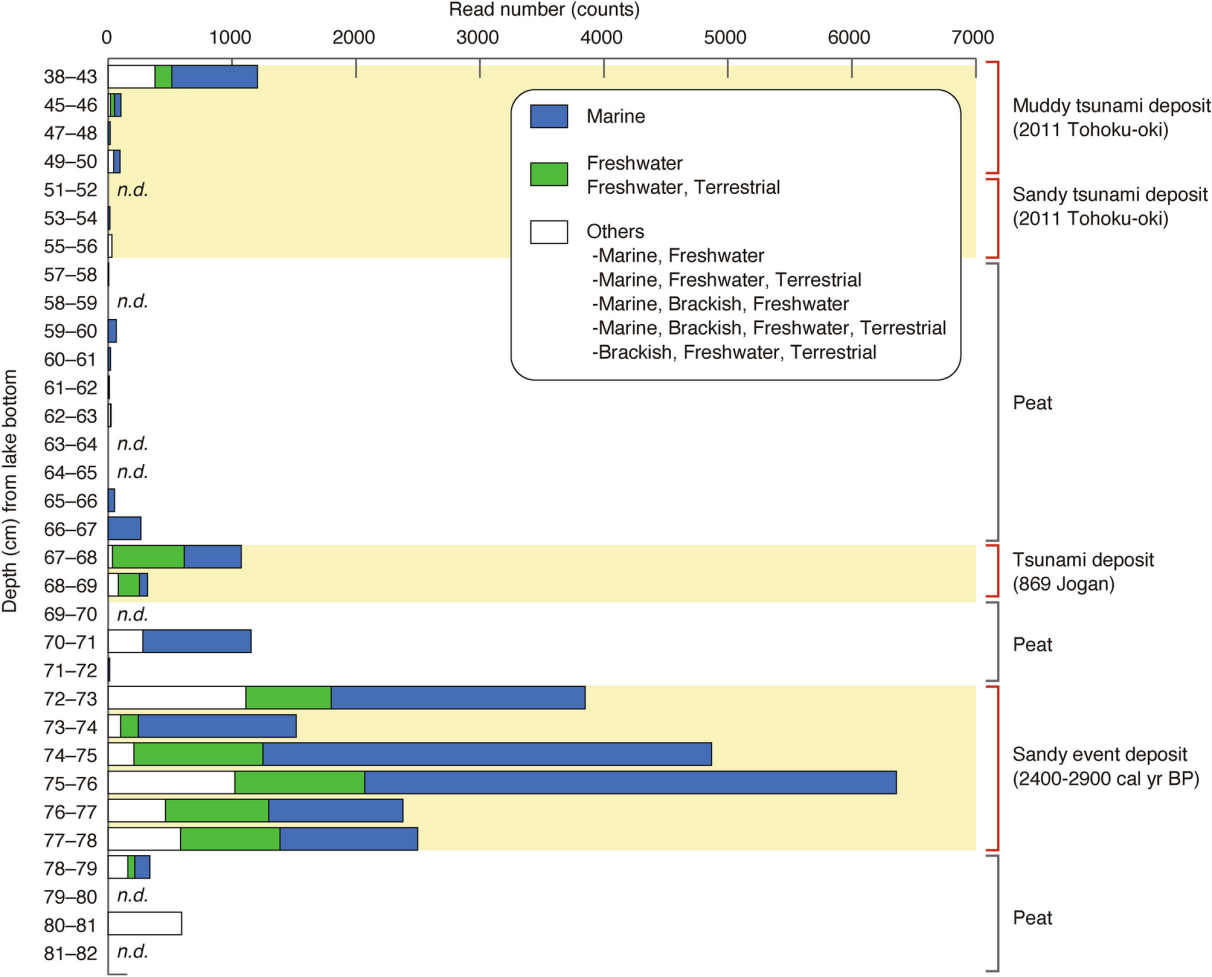
Read number of diatom DNA classified into three salinity groups (marine, freshwater/freshwater and terrestrial, and others). *n.d.* not detected.

*Stramenopiles* and *Chrysophyceae* DNA dominated at 45–66 cm depth (Figs. S2, S3, Table S3). We focused on diatom DNA and identified DNA from marine diatoms (*Nitzschia* spp., *Chaetoceros* spp., *Cyclotella choctawhatcheeana*, and *Gedaniella*) (Fig. 4, Table S4). The marine diatom *Gedaniella* was also detected in the sandy part of the tsunami deposit at 53–54 cm depth, although at a lower abundance than in the muddy layer (Table S4). *Gedaniella* was also present in the layer immediately below the sand layer (57–58 cm depth), suggesting that the diatoms had migrated vertically downward from the sand layer, in which the pore spaces are large (Table S4).

The diverse plant and animal gene sequences found in the muddy part of the tsunami deposit (Figs. 2 and 3) may indicate that debris was transported by the tsunami from the area surrounding the lake. The tsunami deposit analyzed in this study contained DNA from *Pinidae* such as *Pinus*. On the seaward side of the studied lake, black pine trees were planted as tide protection forests in the coastal area before the 2011 tsunami, and their DNA was probably moved to the lake by the tsunami. Thus, the presence of DNA from a variety of plant and animal species that inhabit the surrounding area indicates that the DNA was transported by some high-energy event, and may be a suitable criterion for identifying tsunami deposits. However, DNA was poorly preserved in the sandy part of the tsunami deposit, possibly as a result of DNA decomposition in the oxidative environment of the sand layer. The degradation of short DNA and RNA fragments in the environment is mainly due to hydrolysis and oxidative damage^24^. Environmental DNA in the ancient environment is also referred to as sedimentary ancient DNA (sedaDNA)^25,26^, and in previous studies, the physicochemical properties of the sediment were thought to play an important role in the preservation of sedaDNA^26^. It has been experimentally demonstrated that clayey sediments have high DNA content under low-temperature and anoxic conditions^27^, and that clay enhances the DNA adsorption capacity of the sediment^28^. DNA adsorption to sand is also known^29,30^. In our previous study on extant sand samples, sufficient amounts of DNA have been extracted even from sand and metabarcoding analyses have been successful^31^, but the degradation of DNA samples in ancient sand samples over time would be considerably progressive. There is also some debate that the binding of DNA is weaker in sand than in other sediment types^27^. The relationship between the characteristics of seabed sediment and the preservation of sedaDNA over time is not well understood^26^, and this is an issue for the future. However, the presence of small amounts of DNA derived from marine organisms in the sandy part of the tsunami deposit indicates that this sediment contains allochthonous genetic information.

### Preservation potential of eDNA in paleo-tsunami deposits

As described above, this study has shown that eDNA is an effective proxy for tsunami deposit identification for the 2011 Tohoku-oki tsunami. Next, we examined whether eDNA can be used to identify the event deposits of the 869 CE Jogan tsunami (sandy deposit at 67–69 cm depth), a prehistoric sandy event deposit (72–78 cm depth), and underlying and overlying peat deposits formed under background conditions (69–72 cm and 78–82 cm depth). Regarding *Viridiplantae*, the prehistoric event deposit showed a diversity of taxa similar to that observed in the 2011 Tohoku-oki tsunami deposit, although the Jogan tsunami deposit yielded a lower diversity (Fig. 2, Table S1). Among *Metazoa*, marine bivalves were detected in the Jogan tsunami deposit, and marine bivalves, *Nematodes*, and the sea urchin *Echinodermata* were detected from the prehistoric event deposit (Fig. 3, Table S2). Diatom taxa such as *Chaetoceros* spp. and *Cyclotella choctawhatcheeana* were detected from both the Jogan tsunami and the prehistoric event deposits (Table S4). Diatoms, including some freshwater species, were generally abundant in the tsunami deposits (Fig. 4, Table S4).

The source of the sand of the prehistoric event deposit (72–78 cm depth) has not been assessed, but eDNA analysis indicates that it contains DNA from marine organisms, so it is likely that it was transported by currents from the ocean. At this point, we cannot rule out the possibility that the event deposit was formed by a storm event rather than by a tsunami. The estimated age of deposition of the event sand layer is 2400–2900 cal year BP^20^, so if it was indeed deposited by a tsunami, it may correspond to the tsunami event that occurred in 500–400 BCE in the central part of the Japan Trench^32^ or it could be the event that occurred between 488 BCE and 215 CE in the southern part of the Japan Trench^33^. Regardless, the eDNA results suggest that, even though the event occurred more than 2000 years ago, the event deposit preserves a diverse DNA assemblage that includes material from marine organisms.

**Fig. 5:**
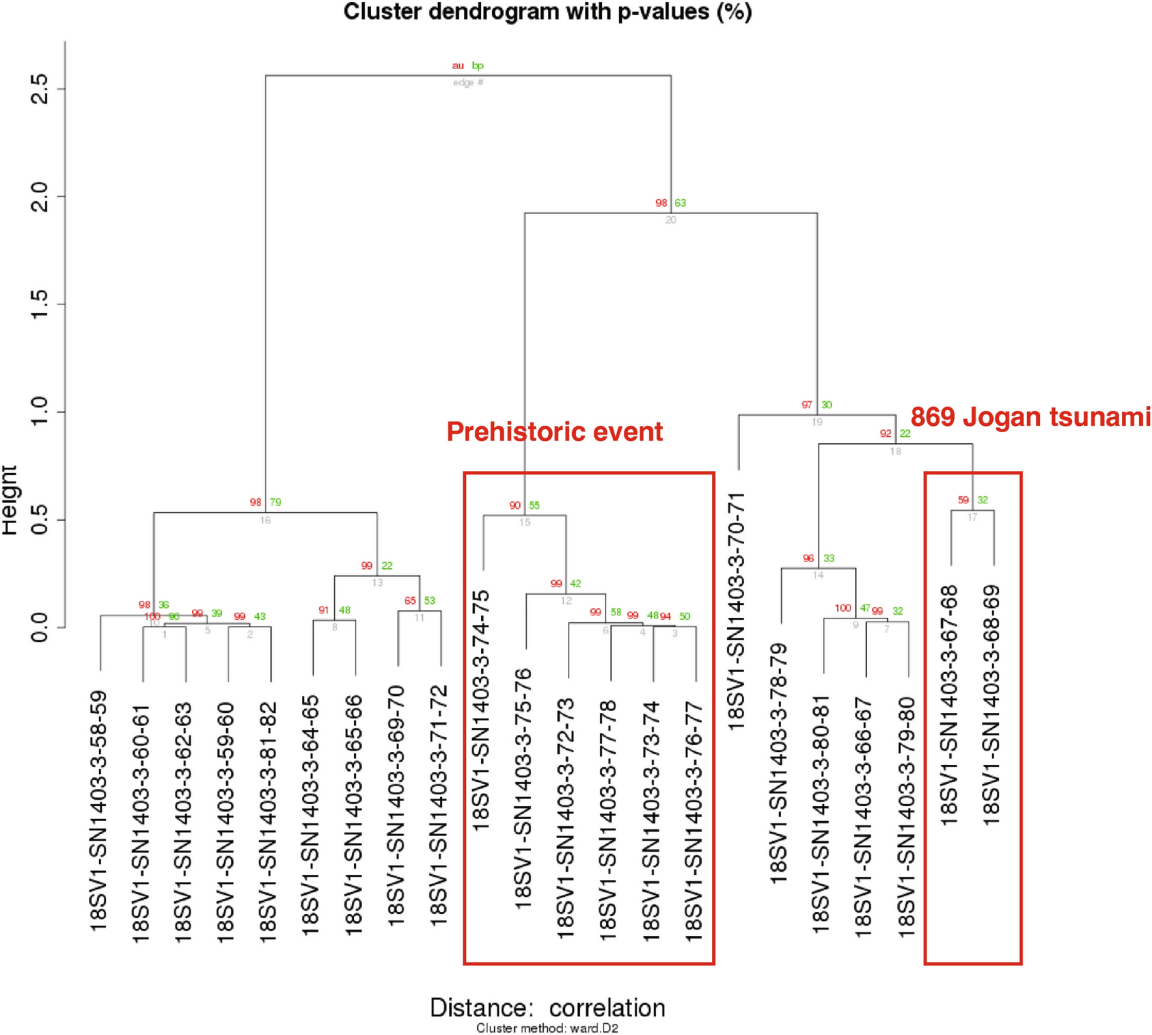
Cluster analysis produced from the results of DNA analysis for 58–82 cm depth in Slice 3. The red numbers at nodes represent the approximately unbiased *p*-values; the green numbers represent the bootstrap probability values.

Cluster analysis of the DNA assemblages contained in each stratigraphic level showed that the Jogan tsunami deposit, the prehistoric event deposit, and the background peat could be divided into different classes (Fig. 5). The difference between the Jogan and prehistoric event deposits may be due to differences in the respective depositional environments. Our results objectively demonstrate that the biological communities preserved in each deposit are different, indicating that eDNA analysis is effective for identifying tsunami deposits, even for historical and prehistoric events.

**Fig. 6:**
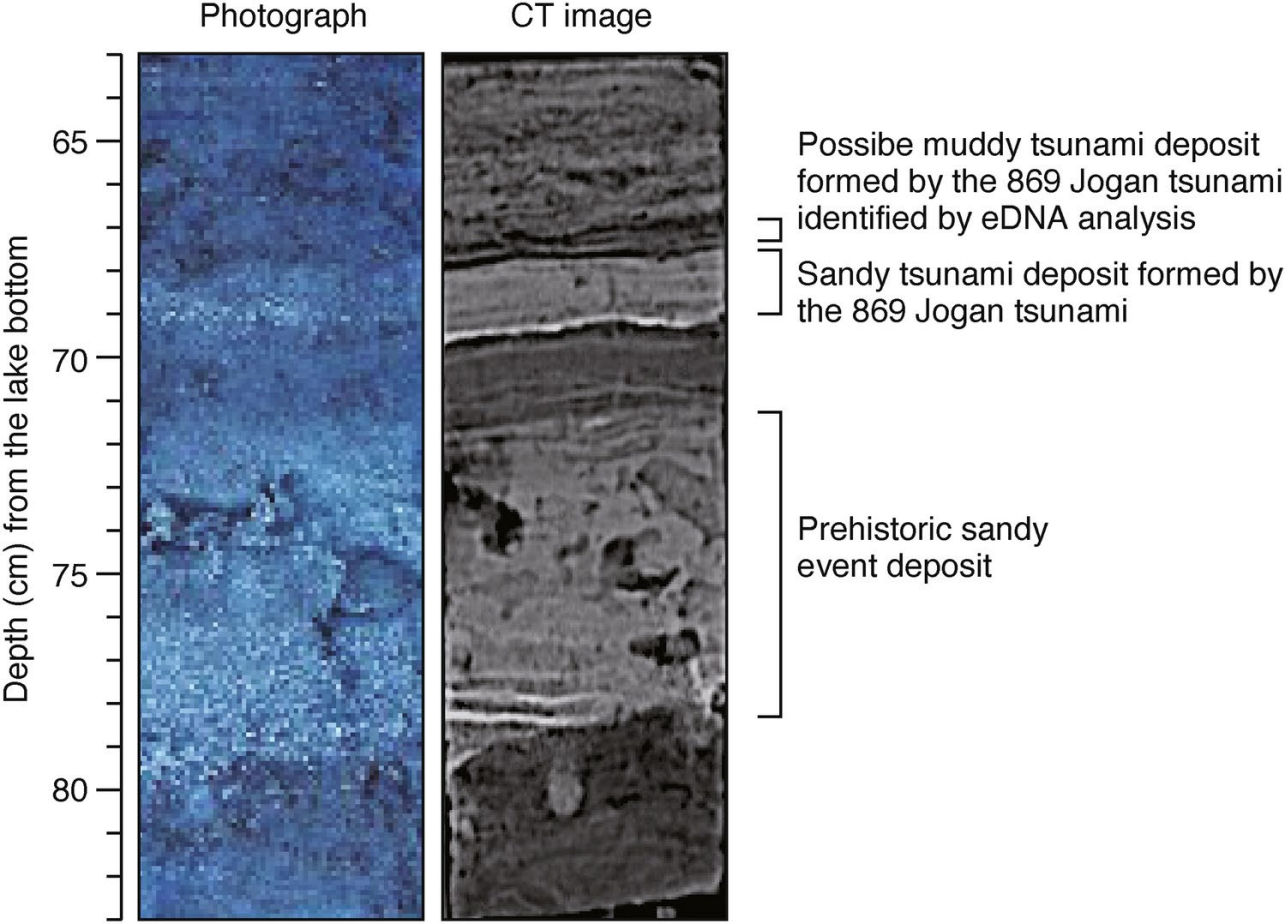
Photograph and CT image of the 63 to 83 cm depth interval in Slice 3.

*Viridiplantae* (*Nymphaeales*, *Saxifragales*, asterids, rosids, *Liliopsida*, *Pinidae*, and *Sphaeropleales*: Table S1) were abundant in both the Jogan tsunami deposit and in the stratum immediately above the tsunami deposit (66–67 cm depth) (Table S1). Although the layer, 66–67 cm depth, could not be distinguished from the overlying peat by either the naked eye or in computed tomography (CT) images, the eDNA results suggest that it is most likely a muddy tsunami deposit that was laid down directly above the sandy tsunami deposit. In the CT image, a thin low-density layer with a laminar-like sedimentary structure is visible directly above the Jogan tsunami deposit layer (Fig. 6). This type of thin mud layer immediately overlying a sandy tsunami deposit is called a “mud cap”, and is a common sedimentological characteristic of tsunami deposits^34^. At Lake *Suijin-numa*, a muddy tsunami deposit was observed directly above the sandy tsunami deposit formed by the 2011 Tohoku-oki event (Fig. 1d). It is inferred that first the sand was deposited by the tsunami wave that entered the lake, and that subsequently the mud suspended in the water slowly settled out and covered the sand. Identification of muddy tsunami deposits in the geological record by visual observation can be difficult, however, because these deposits appear similar to peat or background sediment. In this study, the diversity of eDNA in the muddy deposit from the 2011 Tohoku-oki tsunami and the high abundance of taxa in the layer immediately above the Jogan sandy tsunami deposit indicate that eDNA can be applied to identify muddy tsunami deposits. However, as in the case of the Jogan tsunami deposit, some taxa may not be detected if continuous measurements are not obtained. Isolated measurements, for example, measurement of one point in the event layer and one point in the background sediments, are not sufficient; rather, continuous measurements should be obtained over at least the interval from immediately above to immediately below the event layer.

### Implications for identification of deposits formed by other large tsunami events

**Fig. 7:**
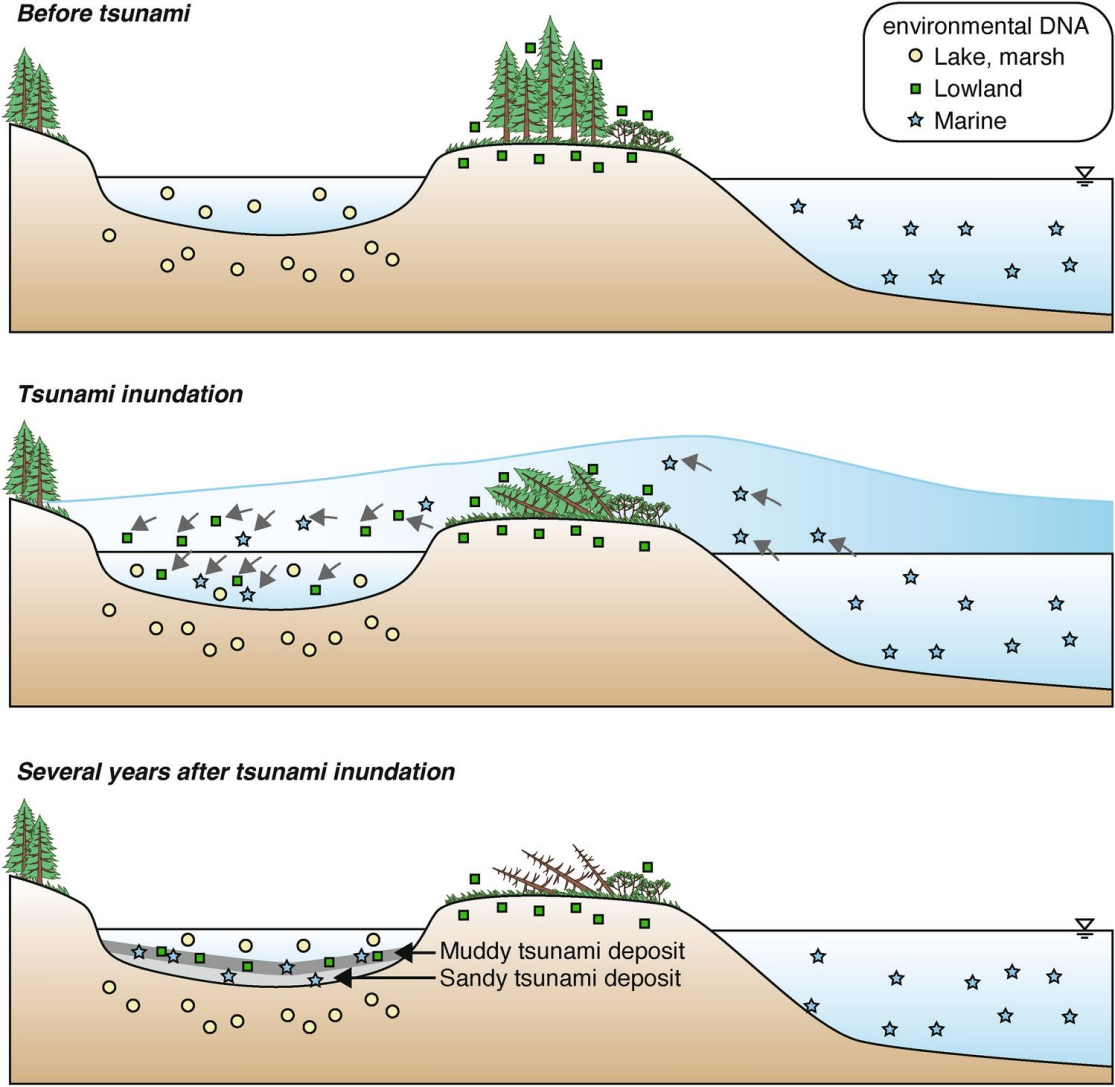
Conceptual model of eDNA transport and deposition by a tsunami.

The results of this study indicate that eDNA can be applied to identify the traces of modern, historical, and prehistoric tsunami events that affected lakes. The use of eDNA has the potential to clarify the origin of event deposits that have been difficult to identify and thus should be applied to various depositional environments in the future. Because DNA from marine organisms is not usually found on land, the presence of marine DNA, in even small amounts, is an extremely reliable indicator of strong flow from the ocean. Lakes and marshes are more depressed than the surrounding landforms, meaning that eDNA can be transported into the lake by a tsunami from the surrounding area but will not readily be transported out again (Fig. 7).

Because DNA is poorly preserved in oxidizing environments^24^, it was detected in low amounts in the sandy tsunami deposits but in larger amounts in the muddy layer (Tables S1–S3). This property may be applied to identify muddy tsunami deposits. In general, it is extremely difficult to distinguish muddy tsunami deposits in the geological record from background deposition; however, it may be possible to identify muddy tsunami deposits by eDNA analysis, as in the case of the muddy layer immediately above the Jogan sandy tsunami deposit (Fig. 6). If tsunami traces that are difficult to detect in the geological record, such as muddy tsunami deposits, can be identified, more accurate estimates of tsunami recurrence intervals could be obtained. Furthermore, it is known that visible tsunami deposits (= sandy tsunami deposits) may not reach the tsunami run-up limit when the tsunami inundation distance is longer than 3 km, leading to the problem that only the minimum tsunami magnitude can be estimated from the distribution of visible tsunami deposits^35^. In such cases, if characteristic eDNA can be detected beyond the visible distribution limit of the tsunami deposit, the tsunami inundation area can be reconstructed with higher accuracy, which in turn will lead to a more accurate estimation of the magnitudes of huge earthquakes and tsunamis. Several recent studies, using methods such as elemental analysis^36–38^ and biomarker analysis^39,40^, have reported evidence for tsunami inundation beyond the distribution of sandy tsunami deposits. In the future, eDNA analysis should also be applied to reconstruct accurate tsunami inundation areas.

The study of tsunami deposits using eDNA is still in its infancy. There are still many issues to be considered, such as what kinds of depositional environments are suitable for eDNA preservation. As mentioned earlier, it is known that DNA is easily degraded and not preserved in oxidizing environments^24^, but the diatom results in this study showed that the number of reads was significantly larger in the 72–78 cm depth prehistoric sandy tsunami deposits than in the sandy tsunami deposits of the 2011 Tohoku-oki tsunami (51–56 cm depth) and the 869 Jagan tsunami (67–69 cm depth) (Fig. 4). Although it is difficult to determine the reason for the observed differences, it is possible that the layers of tsunami deposits were packed by the deposited sand and mud in the tsunami, and that they were preserved under anoxic conditions at once, or that the difference in DNA preservation was caused by the difference in the depositional environment at the time of the tsunami.

In areas of high biological activity, evidence may be lost over periods as short as a few thousand years; however, Willerslev et al.^41^ have reported that plant and animal DNA in soils as old as 400,000 to 100,000 years ago is preserved in permafrost in Siberia and temperate caves in New Zealand. Although the present study was conducted on lake sediments, eDNA preservation should be examined in a variety of sedimentary environments, and also using other DNA markers such as rbcL targeting plant species^42^. In addition, as we focused on detecting small amounts of DNA this time, we carried out metabarcoding analysis using specific primer pairs, but in the future, we would also like to apply shotgun sequencing, which can detect all genomic fragments preserved in sediments without being dependent on length or involving primer bias^26^. In addition, once the genome information of major species is available, it will be useful to use methods such as mapDamage^43^ to verify whether the sequence data obtained from the sediment is ancient DNA or not.

## Methods

Sediment samples were frozen at − 20 °C after excavation, except for some samples that were cut for analysis. After cutting off the periphery of the sediment sample, the sediment was cut into 1 cm thick pieces. When cutting the sediments, gloves were worn, and the knife was wiped with sodium hypochlorite each time it contacted the sample to avoid contamination. A total of 32 layers were analyzed for environmental DNA. To avoid contamination as much as possible, following the eDNA guideline paper^44^, separation of the laboratory before and after PCR, use of a clean bench, etc. DNA was extracted from the sediments by using the DNeasy PowerMax Soil Kit (QIAGEN), and polymerase chain reaction (PCR) analysis was performed on eukaryotic 18S ribosomal RNA gene partial sequences. The basic molecular experiments followed the methods used in our previous study^45^. We targeted the 18S rRNA gene V1–V2 region, including overhang adaptor sequence for MiSeq analysis (F04: 5′-GCTTGTCTCAAAGATTAAGCC-3′^46^; R22mod: 5′-CCTGCTGCCTTCCTTRGA-3′^47^). We also added negative controls to the PCR reactions to check for contamination in a series of experiments. Each sample DNA were amplified in 3 replicate PCR reactions, and mixed triplicate PCR amplicon for each sample into a single tube. The obtained PCR amplification products were purified by magnetic beads (Agencourt AMPure XP Kit: Beckman Coulter, Brea, USA) and a second round PCRs was undertaken to add the index sequence to distinguish each sample in MiSeq analysis. After magnetic beads purification, the PCR products were analyzed by using a next-generation sequencing (MiSeq, Illumina, Los Angeles, CA, USA), and representative sequence determination was performed on the obtained sequences with QIIME2^48^ and amplicon sequence variants (ASVs) were available. Taxon estimation was then performed by homology search of representative sequences of each ASV by BLAST+^49^ against the National Center for Biotechnology Information (NCBI) nucleotide database. Only sequences assigned to each taxon (> 97% similarity) and with longer comparative base lengths (> 200 bp) were used in subsequent statistical analyses. Cluster analysis was performed through the software R package “pvclust”^50^ based on ASV table summarizing genus level (level 6 in QIIME2 output file). Raw data of sequencing was submitted to the DNA Data Bank of Japan (DDBJ), and can be referenced using accession number PRJDB19789.

## Supplementary Information

Supplementary Information 1.

Supplementary Information 2.

Supplementary Information 3.

Supplementary Information 4.

Supplementary Information 5.

Supplementary Information 6.

Supplementary Information 7.

Supplementary Information 8.

## Data Availability

The datasets generated during and/or analyzed during the current study are available from the corresponding author on reasonable request.
