# Supplementary material for: Identification of deposits from modern and ancient large tsunamis by means of environmental DNA
Source: Sci Rep. 2025 Jan 2;15:242. doi: 10.1038/s41598-024-84245-y (PMC11696876; doi:10.1038/s41598-024-84245-y)
Supplement: Supplementary file 1 — Supplementary Information 1. [file 41598_2024_84245_MOESM1_ESM.pdf]

Supplementary Information

Figure S1. Relative abundances of DNA sequences for all eukaryotes in each sampled layer in Slice 3.

Figure S2. Relative abundances of *Stramenopiles* DNA sequences, including *Chrysophyceae*, in each sampled layer in Slice 3.

Figure S3. Relative abundances of *Stramenopiles* DNA sequences, excluding *Chrysophyceae*, in each sampled layer in Slice 3.

Table S1. Number of reads assigned to *Viridiplantae* in each sampled layer in Slice 3.

Table S2. Number of reads assigned to *Metazoa* in each sampled layer in Slice 3.

Table S3. Number of reads assigned to *Stramenopiles* excluding *Chysophyceae* in each sampled layer in Slice 3.

Table S4. Number of reads assigned to diatoms (*Bacillariophyta*) in each sampled layer in Slice 3 and the habitat of each taxon.
