# Supplementary figures and images for: Identification of deposits from modern and ancient large tsunamis by means of environmental DNA
Source: Sci Rep. 2025 Jan 2;15:242. doi: 10.1038/s41598-024-84245-y (PMC11696876; doi:10.1038/s41598-024-84245-y)

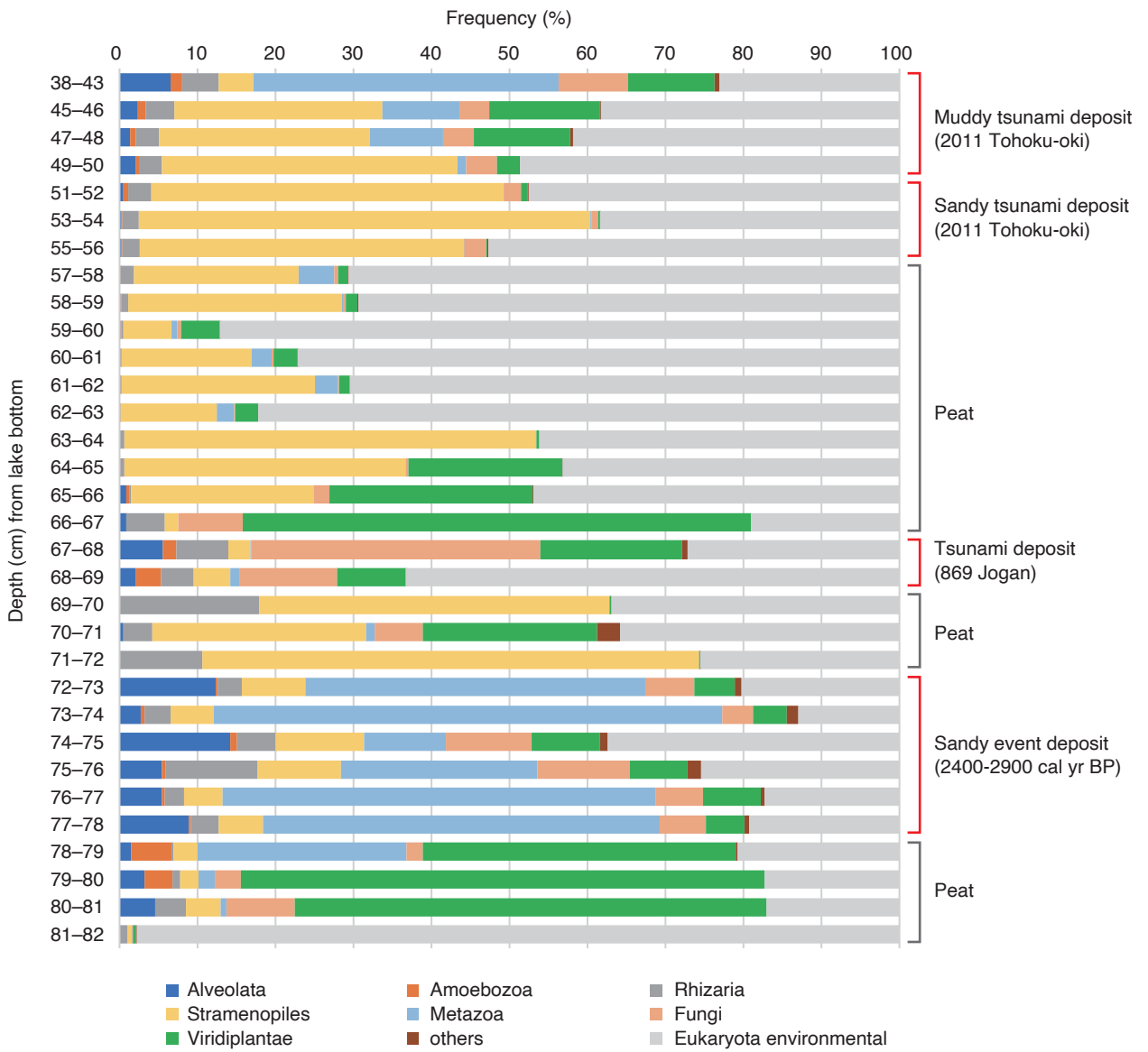

Supplement: Supplementary file 2 — Supplementary Information 2. [file 41598_2024_84245_MOESM2_ESM.pdf]

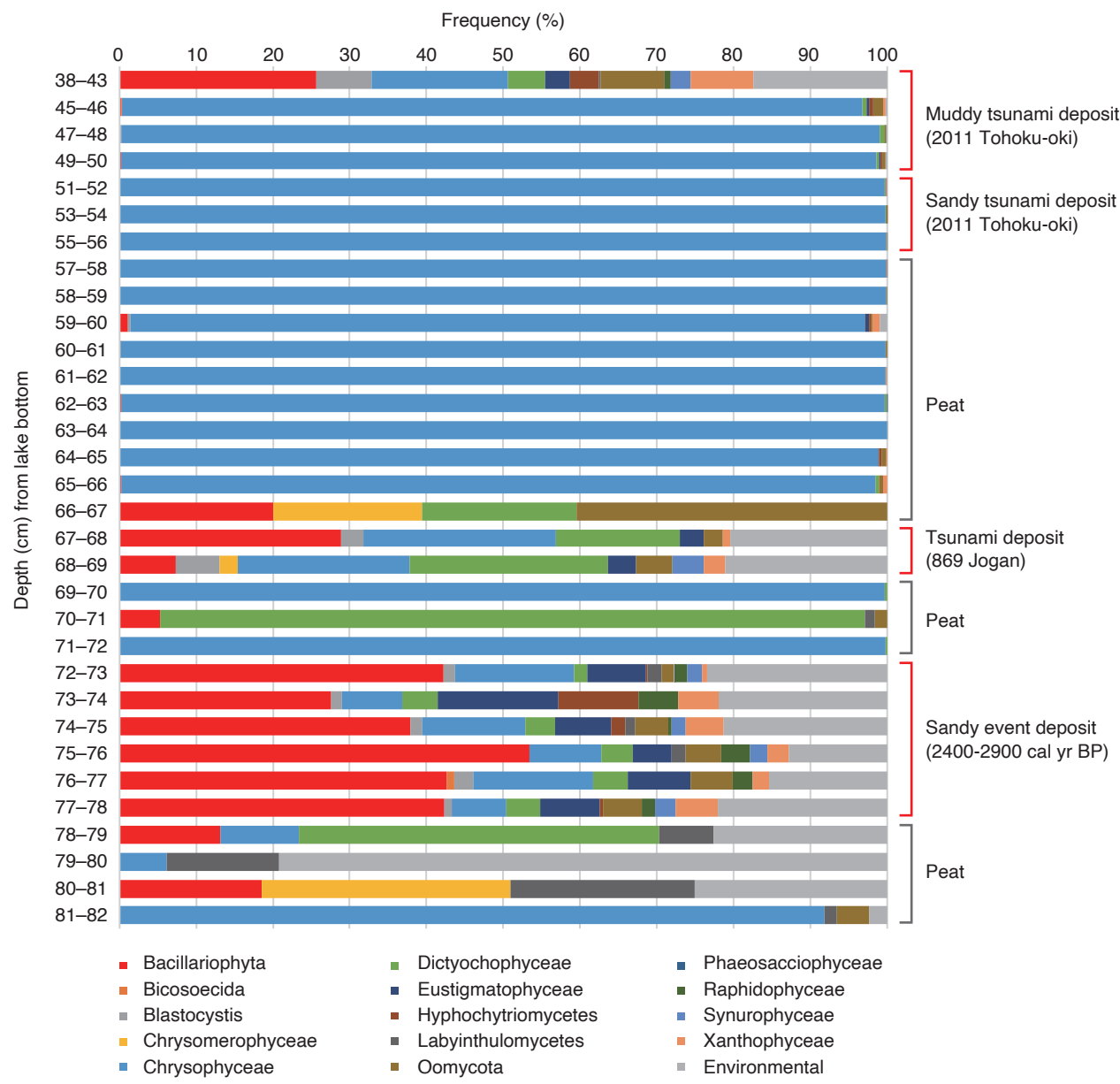

Supplement: Supplementary file 3 — Supplementary Information 3. [file 41598_2024_84245_MOESM3_ESM.pdf]

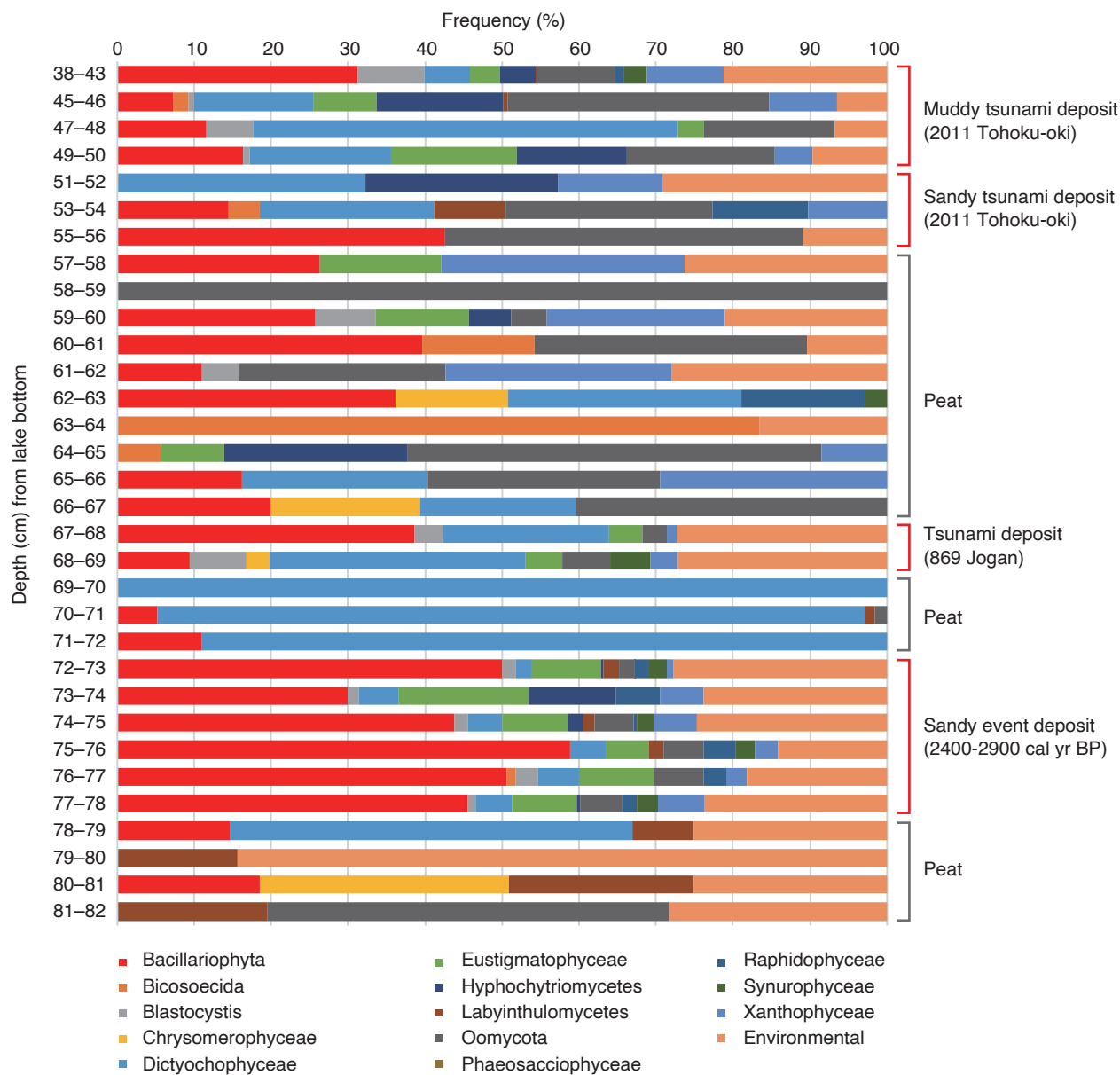

Supplement: Supplementary file 4 — Supplementary Information 4. [file 41598_2024_84245_MOESM4_ESM.pdf]
